# Knowledge, Attitudes, Practices, and Awareness Levels Among Indian Postmenopausal Women About Osteoporosis and Its Relationship With Sociodemographic Factors: A Cross-Sectional Study From Northern India

**DOI:** 10.7759/cureus.59606

**Published:** 2024-05-03

**Authors:** Amit Kale, Nitish Khandelwal, Bhavya Sirohi, Omna Shaki, Sanjay Rai

**Affiliations:** 1 Orthopaedics, Military Hospital, Jammu, IND; 2 Pathology, Military Hospital, Ambala, IND; 3 Orthopaedics, Military Hospital, Agra, IND; 4 Orthopaedics, Command Hospital, Udhampur, IND; 5 Trauma and Emergency, Military Hospital, Ambala, IND; 6 Orthopaedics, Military Hospital, Ambala, IND

**Keywords:** behavior, practices, knowledge, attitude, menopause, osteoporosis

## Abstract

Background

Osteoporosis is a silent disease and can be prevented by providing correct and appropriate information to the individuals at risk. Therefore, we aim to find out the levels of knowledge, attitudes, and behaviors of postmenopausal women, the highest-risk group.

Methods

Between May 2021 and December 2023, a cross-sectional study was done in the Military Hospital in Ambala, India, in 1326 postmenopausal women of age between 45 and 70 years who visited the outpatient department of orthopedics and who previously had a measure of bone mineral density (BMD) or estimation of BMD. All patients participating in the study received a questionnaire that assessed the level of knowledge, attitudes, and behaviors related to osteoporosis. The Osteoporosis Knowledge Assessment Tool (OKAT) was used to assess these parameters.

Results

The mean age was 60±3.1 years. The mean score for osteoporosis awareness was 6/20 points. A total of 983 (73.13%) had no knowledge about osteoporosis, and 221 (16.66%) had higher knowledge. Knowledge about osteoporosis was low with a mean knowledge score of 7.44±3.16 and a median of 7.2. It was found that those who graduated had some knowledge regarding osteoporosis with the help of either a hospital staff or a family member who had a history of osteoporosis.

Conclusions

Even among postmenopausal women who know they are at risk and should have BMD, knowledge, attitudes, and behaviors about osteoporosis were found to be quite low. In addition, education and mass communications are needed to increase awareness among women of this age about improving bone health.

## Introduction

Osteoporosis, which is a silent and common metabolic bone disease, is known by decreased bone mineral density (BMD) with increased chances of fragility fracture, due to the deterioration of bone microarchitecture. Because osteoporosis becomes more common with age, osteoporotic fragility fractures occur more in women than in men after the age of 50 [1]. The worldwide burden or the prevalence of osteoporosis among people aged 15-105 years is estimated to be 18.3% [2]. Osteoporosis is an asymptomatic disease until fragility fractures occur with insignificant injury [3]. These fragility fractures lead to increased morbidity and mortality especially if they occur in the hip region. Considering the increasing number of elderly population, the problem of osteoporosis is increasing every day. Therefore, it is very important to identify factors responsible for the development of osteoporosis. This can be achieved by obtaining knowledge about osteoporosis, cultivating self-efficacy, implementing essential lifestyle modifications, and fostering preventive measures through increased awareness and timely diagnosis [4]. The issue of osteoporosis and its associated disorders has garnered significant discussion among healthcare givers and researchers due to its public health and economic implications. Given these factors, the avoidance of this disease can substantially decrease the expenses of the healthcare system [5].

Although osteoporosis knowledge, attitude, and awareness have been demonstrating a favorable impact on the prevention of osteoporosis in developing countries like India, the study in this regard especially in the Indian women population is insufficient. Therefore, this study aimed to clarify the gaps by examining the knowledge, attitudes, and behaviors of postmenopausal patients presenting to a tertiary care hospital for osteoporosis.

## Materials and methods

Study design and setting and duration

A cross-sectional study was undertaken in the Military Hospital in Ambala, India, to assess the knowledge, attitudes, and awareness levels among Indian postmenopausal women about osteoporosis between May 2021 and December 2023. Approval was obtained from the Ethical Committee of the Military Hospital in Ambala (approval number: MAH/Ortho/Jan 2020). All included postmenopausal women were given a researcher-designed questionnaire that assessed their knowledge, attitudes, and behaviors about osteoporosis. Questions were distributed in the study based on positive and negative behaviors to assess the association with osteoporosis. Regular exercise, calcium intake, and sun exposure were considered positive, while negative behaviors included smoking, alcohol consumption, a sedentary lifestyle, and dietary errors.

Participants

Sample Size and Inclusion Criteria

There were a total of 1397 postmenopausal women aged between 45 and 70 years who visited the outpatient department of orthopedics. Seventy-one women who did not satisfy the inclusion criteria were excluded from the study; hence, 1326 postmenopausal women who visited the said department and who previously had a measure of BMD or estimation of BMD were included in the study. Women were excluded if they previously had BMD measured; had hypo- or hyperthyroidism, malignancy, chronic kidney diseases, or autoimmune/inflammatory joint disease such as systemic lupus erythematosus (SLE) and rheumatoid arthritis; were hysterectomized; and had received hormone replacement therapy.

Data collection

A pre-designed semi-structured questionnaire consisting of 20 items, with response options of true, false, and don't know, was provided to the women enrolled in the study. In addition, face-to-face interviews were done to collect data. The interview of each postmenopausal woman took an average of 18 minutes. The initial segment of the questionnaire included sociodemographic data that included personal details, education, habitat, body mass index, weight, family, and socioeconomic status from Kuppuswamy's socioeconomic scale. The second section included educational level and lifestyle behavior history. The third section included the level of physical activity and diet. Questions included in the study included positive and negative behaviors to assess the association with osteoporosis. Regular exercise, calcium intake, and sunbathing were considered positive, while negative behaviors included smoking, alcohol consumption, a sedentary lifestyle, and dietary errors. The Osteoporosis Knowledge Assessment Tool (OKAT) was used to assess these parameters [6-8].

The OKAT is a 24-item test. The first nine items evaluate the general knowledge of osteoporosis risk, the next eight ask about calcium, and the final seven ask about activity. The widespread usage of this Kim et al. [6] instrument, which has internal reliability values of 0.69 for OKAT exercise (items 1-16) and 0.72 for OKAT calcium (items 1-9 and 17-24), supports its strong validation [7]. The total number of right answers was added together to determine each person's score.

Knowledge from the OKAT questionnaire

Knowledge-related questions included understanding of bone health and osteoporosis and its prevention, fragility fracture, and personal habits. Twenty questions were asked to assess the level of osteoporosis knowledge of the study participants. The state of the individuals' knowledge was assessed based on the responses provided to the questions. The mean knowledge score was 7.44±3.16, and the median was 7.2. The level of knowledge was rated as high (75%), medium (50-75%), and low (less than 50%).

Attitudes from the OKAT questionnaire

The mean attitude score was 11.52±3.31, the median was 9, and 724 (54.60%) had a low or negative attitude. Considering the behaviors of the participants of the study towards osteoporosis, 76.99% of them did not smoke, and 69.68% did not drink alcohol. About 52.18% of women exercised sometimes, 34.53% walked mostly inside their houses, and 31.07% exercised for less than 30 minutes. Around 33.33% of women used calcium preparations and 29.33% used vitamin D supplements. About 24.96% of women in the study were exposed to the sun every day. Around 70.28% of women usually take 1-2 servings of milk or milk products daily, and 1.5% of women were taking medication for other medical problems that lower BMD.

The attitude was rated as positive (75%), neutral (50-75%) and negative (less than 50%). Postmenopausal women with an attitude score of <50% were described as having a negative attitude, a score of 50-75% was considered neutral, and a score of >75% was considered positive.

We evaluated individuals according to their unique behaviors related to osteoporosis, geographical region, and previous awareness of osteoporosis. Women with higher levels of knowledge exhibited a greater propensity for smoking compared to those with lower levels of knowledge. People who took sun exposure regularly every day had a higher knowledge of osteoporosis. Similarly, people consuming 3-4 portions of milk and milk products per day had a significantly higher level of knowledge.

Osteoporosis awareness from the OKAT questionnaire

The average accuracy rate for answering the questions was 29.3% (7.4/20). Approximately 71% of the women exhibited a concerning lack of knowledge among the overall replies. The study group's level of awareness was described as follows: good, 5.3%; average, 34.1%; poor, 38.1%; and very poor, 22.5%.

Exercises from the OKAT questionnaire

Self-reported questions were related to the physical exercise level like outdoor walking, sports, jogging, gym, and aerobics. Each question was scored 1 for good practice and 0 for inappropriate practice. These questionnaires are the most common performance assessment method [7] and are based on the recall ability of participants.

The level of activity was assessed as good (75%), sufficient (50-75%), and insufficient (<50%). Women who scored less than 50% were considered to have an inadequate level of exercise, while those who scored between 50% and 75% were considered to be exercising at a good level.

Sample size calculation

To estimate the sample size, the prevalence was taken as 50%, the confidence level was 95%, and the margin of error (d) was set at 5% (e.g., alpha=0.05).

Statistical analysis

The data analysis was conducted utilizing IBM SPSS Statistics for Windows, Version 21.0 (Released 2012; IBM Corp., Armonk, New York, United States). We investigated the association between attitude levels and their knowledge and variables through the utilization of the γ2 test. Additionally, we also examined the relationship between estimated scores and food consumption frequency by Pearson's correlation test. Furthermore, the disparities in scale scores for demographic variables using independent group t-tests and ANOVA tests were also analyzed.

## Results

The average age of the women was 60±3.1 years, and the average body mass index was 29.53±5.06 kg/m^2^. About 19% of the women had higher education, and 56.78% completed primary school. Around 43.58% of them were housewives. About 89.29% of the women had osteoporosis in the family, and only 25.86% had previously had some knowledge regarding osteoporosis. Most (25.7%) information about osteoporosis was gained from the mass media, such as TV, newspapers, and the internet (Table 1).

**Table 1:** Sociodemographic parameters and osteoporosis information sources of the women *: verified by medical document (women were unaware about osteoporosis)

Parameters	N (%) N=1326
Age (mean SD)	60±3.1
Weight (mean SD) in kg	45.20±18.77
Height (mean SD) in feet	4.21±1.54
Level of education	
Primary education	753 (56.78)
High school and higher secondary	321 (24.2)
University (graduation and above)	252 (19.0)
Work profile	
Office worker	252 (19.0)
Housewife	578 (43.58)
Manual laborer	102 (7.69)
Retired	394 (29.71)
Status of getting information about osteoporosis	
Yes	343 (25.86)
No	983 (73.13)
Source of information about osteoporosis	
Received	343 (25.86)
By media like newspaper/TV/internet	98 (7.39)
By healthcare personnel	135 (10.18)
By neighbor/relative/friend	110 (8.29)
Not received at all	983 (73.13)
Presence of osteoporosis in the family	
Yes	1184 (89.29)*
No	142 (10.70)*

Twenty questions were asked to assess the level of osteoporosis knowledge of the study participants. The state of their knowledge was assessed by answers given by the women to these questions (Table 2). The average knowledge score was 7.44±3.16, and the median was 7.2. Nine hundred and eighty-three (73.13%) women had a low level or no knowledge. Upon analyzing the osteoporosis information scores derived from the questionnaire, it was found that individuals with a family history of osteoporosis, those with a university education, those who had received prior information about osteoporosis from a doctor, and individuals below the age of 45 had significantly higher average scores compared to others.

**Table 2:** Comparing the knowledge scores of women on osteoporosis based on their demographic features *: verified by medical document (women were unaware about osteoporosis)

Parameters	Scores	F	P-value
Age (year)		2.822	0.043
≤45 (n=198)	8.45±4.48		
46-55 (n=622)	6.77±4.65		
56-65 (n=365)	7.65±3.76		
≥66 (n=141)	5.98±3.59		
BMI (kg/m^2^)		2.638	0.029
Normal weight (18.5-24.9)	8.23±4.11		
Overweight (25.0-29.9)	7.2±4.00		
First-degree obese (30.0-34.9)	5.79±3.95		
Second-degree obese (35.0-39.9)	7.41±4.69		
Third-degree obese (≥40)	8.69±5.09		
Level of education		4.979	0.001
Primary education	753 (56.78)		
High school and higher secondary	321 (24.2)		
University (graduation and above)	252 (19.0)		
Number of children		0.236	0.682
1-3	6.43±2.01		
>3	1.03±2.69		
Monthly household income/months in INR		2.692	0.052
<15000 INR	7.27±3.86		
15001-45000	8.45±4.12		
45001-75000	9.41±4.73		
>75000	11.71±6.54		
Socioeconomic class		3.862	0.035
Upper (I) 41 (3.09)	11.34±2.55		
Upper middle (II) 103 (7.76)	10.77±3.01		
Lower middle (III) 397 (29.93)	9.67±3.90		
Upper lower (IV) 153 (11.53)	8.44±5.09		
Lower (V) 632 (47.66)	6.11±2.83		
Chronic disease		−0.387	0.743
Yes	7.54±4.72		
No	7.13±3.89		
Presence of osteoporosis in the family		3.412	0.001
Yes, 1184 (89.29)*	8.4±2.68		
No, 142 (10.70)*	6.55±4.45		
Knowledge about osteoporosis		0.421	0.023
Yes 343 (25.86)	7.26±4.23		
No 983 (73.13)	6.87±4.13		
Source for information on osteoporosis		3.041	0.034
Media like newspaper/TV/internet	8.21±3.09		
Healthcare personnel	7.46±4.24		
Neighbor/relative/friend	5.41±4.67		

The mean attitude score was 8.32, the median was 8, and 52% had a low attitude. Considering the behaviors of the participants of the study to osteoporosis, 69.68% did not drink alcohol, 76.99% of them did not smoke, about 81.3% of the women exercised very occasionally, 34.53% walked mostly indoors, and 58.22% exercised for 30-60 minutes. Around 66.66% of the postmenopausal women did not take any calcium preparations, and similarly 70.66% of the women did not take vitamin D supplements. About 24.96% of the women in the study were exposed to the sun daily, and 31.90% had sun exposure once a month which takes less than an hour. About 70.28% of the women take one or two servings of milk or milk products daily, and 62.06% of the women did not know that the medicine they are taking for their medical problems will lower their BMD (Table 3).

**Table 3:** Summary of daily behavior parameters among women

Variables	N (%)
Alcohol	
<2 glasses a day	12 (0.90%)
Occasionally (social drinking)	390 (29.41%)
No	924 (69.68%)
Smoking	
≥10 pieces per day	13 (0.98%)
<10 pieces per day	82 (6.18%)
Occasionally	210 (15.83%)
No	1021 (76.99%)
Exercising	
≥3 per week	486 (36.65%)
1-2 times a week	692 (52.18%)
Time to time (occasionally)	241 (18.17%)
No	148 (11.16%)
Level of physical activity	
Indoor activity only	458 (34.53%)
Walk (>3 km)	293 (22.09%)
Brisk walking (>2 km)	321 (24.20%)
Running till 1 km	152 (11.46%)
Other (manual labor working in construction site/field)	102 (7.69%)
Daily exercise time	
≥60 min	142 (10.70%)
30-60 min	772 (58.22%)
<30 min	412 (31.07%)
Taking vitamin D supplement	
Yes	389 (29.33%)
No	937 (70.66%)
Taking calcium supplement	
Yes	442 (33.33%)
No	884 (66.66%)
Daily consumption of milk or dairy products	
≥5 servings	10 (0.75%)
3-4 servings	108 (8.14%)
1-2 servings	932 (70.28%)
Hardly ever	276 (20.81%)
Duration of body part exposed to sunlight	
Every day	331 (24.96%)
2-3 times a week	139 (10.48%)
1 time per week	350 (26.39%)
1-3 times a month	423 (31.90%)
Hardly ever	83 (6.25%)
Taking bone mineral density-reducing medications for other medical problems	
Yes	14 (1.05%)
No	823 (62.06%)
Do not know	489 (36.87%)

We assessed women based on specific behaviors related to osteoporosis, location, and previous knowledge regarding osteoporosis. Women with greater knowledge scores smoked more than those who had low knowledge scores. Women who take daily sunshine had a higher knowledge of osteoporosis. The level of knowledge of women consuming 1-2 servings of milk and milk products in a day was also significantly greater.

The Hosmer-Lemeshow test result was 15, and the binominal logistic regression model was statistically significant with a value <0.001. This model predicted an 11.8% variation in Indian women's sufficient comprehension of osteoporosis (Nagelkerke R Square). Three predictor variables knowledge, attitudes, and practices were statically significant (Table 4).

**Table 4:** Overall knowledge, attitudes, and practices among postmenopausal women and binominal logistic regression analysis

Knowledge, attitude, and practices	N (%)	Odds ratio	95% CI (lower-upper)	P-value
Knowledge				
<50% (not knowledgeable)	983 (73.13%)	0.913	0.604-0.662	0.021
50-75% (average knowledge)	122 (9.20%)	0.841	0.583-1.056	0.013
>75% (knowledgeable)	221 (16.66%)	0.8.56	0.564-1.022	0.031
Score (mean±SD)	7.44±3.16			
Attitude				
<50% (negative attitude)	724 (54.60%)	0.823	0.645-0.933	0.021
50-75% (neutral attitude)	421 (31.74%)	0.724	0.667-0.958	0.046
>75% (positive attitude)	181 (13.65%)	0.876	0.782-0.931	0.013
Score (mean±SD)	11.52±3.31			
Practices				
<50% (poor)	807 (60.85%)	0.945	0.833-0.967	0.019
50-75% (fair)	309 (23.30%)	0.891	0.633-0.951	0.017
>75% (good)	201 (15.15%)	0.749	0.791-0.997	0.024
Score (mean±SD)	12.71±9.21			

According to the data shown in Table 5, the average knowledge score was 7.44±3.16, with an interquartile range (IQR) ratio of 4.93. In contrast, the average practice score was 2.1, with an IQR ratio of 4:2. The Spearman rank correlation coefficient was computed to be 0.017, with a p-value of 0.0021.

**Table 5:** Correlation between knowledge and practice based on Spearman rank correlation coefficient IQR: interquartile range

Variable (n=1326)	Mean SD	IQR	Spearman rank correlation	P-value
Knowledge	7.44±3.16	4.93	0.017	0.0021
Practices	12.71±9.21	2.1		

## Discussion

The present study aimed to assess awareness of osteoporosis in postmenopausal women in a teaching hospital in Haryana, India, particularly in relation to risk knowledge, attitude awareness, and practices. Postmenopausal women were selected as study participants because they have a higher chance of osteoporosis and fragility fractures, which requires early and preventive screening in this group.

Our study revealed that 73.13% of postmenopausal women had no knowledge and 16.66% had knowledge about osteoporosis, 31.07% were doing exercise sometimes, and 89.29 had a family history of osteoporosis. It is very important for postmenopausal women to protect themselves against osteoporosis and the complications of the disease. The prevention of osteoporosis by proactive measures is intricately linked to enhancing the knowledge and awareness of the postmenopausal women population.

Osteoporosis is a major public health concern that can be slowed or even prevented, for example, through lifestyle changes. Since it can last a long time without symptoms, possible complications can be prevented if risk factors are suspected at an early stage and a BMD measurement is performed in time. Deficiency of estrogen hormone during pre- and postmenopausal periods leads to a reduction in BMD. The prevalence of osteoporosis among women aged 50 and above in the United States was found to be 15.8% [9]. In our study, the proportion of patients diagnosed with or reported to have osteoporosis was 19.1%, respectively.

In our study, 73.13% of the postmenopausal women had no knowledge about osteoporosis. A review of the literature revealed that similar results were obtained; however, the percentage of knowledge is somewhat varied between 34% and 51% [10-15]. Apart from these studies with insufficient knowledge about osteoporosis, the literature was replaced by other studies with high or moderate knowledge about osteoporosis. Senthilraja et al. in their study reported that 77% of the women had high or moderate knowledge about osteoporosis [16].

Our study yields contrasting findings compared to these studies. There was speculation on the potential correlation between their heightened awareness of osteoporosis and their educational attainment.

In the present study, we recorded a statistically significant difference in osteoporosis data points between women with different education levels. Knowledge about osteoporosis is highest among those women who had graduated but lowest among less educated women.

El-Tawab et al. [17] and Alhouri et al. [18] noted similarly that women with graduation had higher knowledge than those who were less educated. In our survey, participants were asked if they had previously received information about osteoporosis. Only 25.86% of women agreed that they received information.

They have revealed that information regarding osteoporosis was received through television, health workers, and newspapers. The media was the commonest source of information about osteoporosis as reported by many authors [10,13,17,18]. Women with diverse sources of knowledge on osteoporosis scored significantly differently regarding osteoporosis in the present study. Those who learned about osteoporosis from medical experts knew the most about it, while those who learned about it from friends and relatives knew the least. From this vantage point, it is evident that physicians and other healthcare providers must educate and connect with a larger population regarding osteoporosis.

As far as we are aware, this is the first study conducted in north India to gauge postmenopausal women's awareness of osteoporosis. In general, it was discovered that this group lacked knowledge regarding osteoporosis and its risk factors and available treatments. We noted a significant awareness, practice, and knowledge gap about osteoporosis and its prevention; similarly, a study by Gopinathan et al. with 100 postmenopausal women recorded a similar gap among these women [19].

Despite the poor knowledge of those women who took part in our study, only 0.98% smoked, and 0.90% consumed alcohol. This unexpected outcome made us believe that the group with the lowest knowledge score on osteoporosis was actually helping them in the prevention of osteoporosis, but they were unaware of it. An osteoporosis knowledge assessment questionnaire was provided to postmenopausal women in this study, and the findings revealed that 78.92% of the women were unaware that smoking can cause osteoporosis [20]. All of this indicates how challenging it is to educate the public about osteoporosis and change attitudes and behaviors. By preserving existing bone mass in the elderly and maintaining maximum bone mass growth in the young, physical activity helps prevent osteoporosis. While a sedentary lifestyle causes bone loss, regular physical activity has been demonstrated to have a favorable impact on bone health [21-23].

Our study revealed that 18.17% of women exercise occasionally and 11.16% of women do not exercise at all, indicating poor awareness and knowledge about bone health and osteoporosis. Han and Lee [24] and von Hurst and Wham [25], in their study, recorded that participants were found to have low levels of physical activity and were previously uninformed about the advantages of physical activity in the prevention of osteoporosis.

Saltık et al. [13], Vita et al. [26], and Zhao et al. [27] have shown that training and incentives for people to do regular isometric exercises are effective in preventing osteoporosis. In our study, 89.29% of families had a history of osteoporosis among the study population. Many studies have reported that a positive family history increases the chances of developing osteoporosis [16,28-32].

In their study, Senthilraja et al. [16] found that approximately 59% of postmenopausal women were cognizant of the fact that having a family history of osteoporosis enhanced the likelihood of developing osteoporosis. Ahmadieh et al. [14] recorded similar results in more than 50% of perimenopausal and postmenopausal women. Similarly, Koç et al. [12] recorded in 18-35-year-old females that a family history of osteoporosis is a risk factor for the development of the same.

Dietary habits play a vital role in minimizing the development of osteoporosis. A diet deprived of vitamin D (cholecalciferol) and elemental calcium, in these postmenopausal women, causes a decrease in BMD and thus increases the risk of developing fragility fracture. Enough daily recommended doses of calcium and vitamin D can be fulfilled by taking enough milk and dairy products. The International Osteoporosis Foundation (IOF) recommends 1200 mg of calcium per day for postmenopausal women. This requirement must be made by home food. In the present study, 932 (70.28%) of women reported consuming 1-2 servings of milk and milk products. However, 276 (20.81%) women said they hardly ever take milk and milk products. Thus, this habit leads to inadequate calcium intake.

Four hundred and forty-two (33.33%) women were taking calcium supplements, and 389 (29.33%) women were taking vitamin D supplements. Ahadi et al. [33] reported that about 72.2% of women knew the importance of dairy products for good bone health.

Goh et al. [34] in their study noted that 99% of women were taking a diet that was severely deficient in calcium while examining knowledge and behavior about osteoporosis in women with early menopause due to premature ovarian failure.

Consistent with previous research, we discovered a noteworthy correlation between women's knowledge of osteoporosis and their regular intake of milk or milk products. High levels of attitudes and behaviors related to osteoporosis have been observed in those who consume three to four servings of dairy products each day.

A total of 331 (24.96%) and 139 (10.48%) of the study women exposed their bodies to sunlight daily, twice a week, and almost never, respectively. Significant variations in osteoporosis knowledge and attitude were seen among women based on the frequency of sun exposure. Those women who spent their entire day under direct sunlight had the highest degree of knowledge and attitude. In addition, it was revealed that 90.2% of people knew that exposing their body to sunlight is important to protect against osteoporosis.

Nohra et al. [35] noted that 94.4% of participants were unaware of the fact that sunlight is a good source of vitamin D and can influence vitamin D production from the skin. On the contrary, Darout et al. [36] conducted a study including a total of 546 women, of which, 88 (16.1%) were health professionals and 458 (83.9%) were non-health professionals, and noted that 48.1% of health professionals and 44.2% of non-health professionals have less knowledge of osteoporosis behavior, vitamin supplements, and sunlight exposure.

Many studies have shown that the sun on the skin has a favorable effect on bone health through the synthesis of vitamin D [37-39]. Hence, it is crucial to educate individuals about the significance of sunbathing in a proper and sufficient manner for maintaining optimal bone health, considering the time of day and duration. This will help raise awareness and promote the adoption of this activity.

Strengths, limitations, and future goals

The study's strength lies in its pioneering nature as the first hospital-based investigation on knowledge, attitudes, practices, and awareness conducted in an urban location in Ambala, Haryana, India. A suitable formula was utilized to determine the appropriate sample size. All postmenopausal women provided responses to all questions posed during the interview, with no instances of dropout. This can be attributed to the fact that the BMD estimation was conducted at no cost, as the study was carried out in a government-operated hospital.

The study is limited in its generalizability due to its confinement to an urban area in India. The study employed a nonprobability sampling technique, which introduces the potential for recollection bias among postmenopausal women.

To enhance the external validity generalization, it is advisable to reproduce the study. Additionally, conducting a comparative study among rural and urban postmenopausal women will help address the existing disparities in knowledge, attitude, habits, and awareness between the two groups. Moreover, it is possible to conduct a comparable investigation with alternative research methodologies in order to provide insights into the appropriate strategies for addressing osteoporosis, which may not be well-captured by cross-sectional investigations.

Enhancing educational possibilities for women can facilitate their acquisition of knowledge, enabling them to enhance their bone health and mitigate the risk of fragility fractures.

## Conclusions

Based on the findings of our research, it is imperative to enhance public consciousness regarding osteoporosis, a significant concern within the realm of public health.

Preventive medicine has a crucial role in averting the onset of diseases. Promoting societal awareness is the initial stage in establishing factors such as food, exercise, and the advantages of sunlight as integral aspects of one's lifestyle. Enhancing attitudes and actions pertaining to the prevention of osteoporosis poses significant challenges within civilizations characterized by limited information. At this juncture, a significant burden is placed on healthcare professionals, particularly physicians, particularly those specializing in family medicine, who bear the task of delivering preventative healthcare services. Given the consistent and prolonged provision of health services by primary care physicians to a specific community, it becomes evident that they play a crucial role in promoting awareness and fostering a general understanding of osteoporosis.

The most important service possibilities are informing women, conducting protection studies, training and identification of risk groups, and participating in the solution through screening. Today, awareness can be increased by disseminating precise, current, and impactful information that is readily accessible to all individuals through many channels, including radio, television, and the internet. However, it is necessary to compare and verify the accuracy of the information offered by professionals.
